# Economic and disease burden of Japanese encephalitis in Zhejiang Province, 2013–2018

**DOI:** 10.1371/journal.pntd.0009505

**Published:** 2021-06-21

**Authors:** Xuan Deng, Rui Yan, Zi-qiao Li, Xue-wen Tang, Yang Zhou, Hanqing He

**Affiliations:** 1 Zhejiang Provincial Center for Disease Control and Prevention, Hangzhou, People’s Republic of China; 2 Xiamen University, Xiamen, People’s Republic of China; Institute for Health Metrics and Evaluation, UNITED STATES

## Abstract

**Background:**

Japanese encephalitis (JE) is a mosquito-borne disease and associated with high mortality and disability rate among symptomatic cases. In the absence of local data, this study estimated the economic burden and the disability-adjusted life years (DALYs) due to JE in Zhejiang Province, China during 2013–2018, to increase disease awareness and provide evidence for effective health policy.

**Methodology/Principle findings:**

We merged multiple data sources, including National Notifiable Disease Registry System (NNDRS), patient interviews and medical records from corresponding hospitals for JE cases which occurred during 2013–2018 in Zhejiang Province. Direct costs were extracted from hospitals’ billing systems and patient interviews. Indirect costs and disease burden were calculated based on questionnaire survey from patient interviews and follow-up assessment by general practitioners. Given under-reporting, an expansion factor (EF) was applied to extrapolate the JE burden to the provincial level. The total economic burden of JE during 2013–2018 was estimated at US $12.01 million with an EF = 3. Of this, $8.32 million was due to direct economic cost and $3.69 million to indirect cost. The disease burden of JE was 42.75 DALYs per million population (28.44 YLD, 14.28 YLL) according to the 1990 Global Burden of Disease (GBD 1990) methodology and 80.01 DALYs (53.67YLD, 26.34YLL) according to the GBD 2010 methodology. Sensitivity analysis demonstrated that the overall economic burden varied from US$ 1.73–36.42 million. The greatest variation was due to the prognosis of illness (-85.57%-203.17%), followed by occupation (-34.07%-134.12%) and age (-72.97%-47.69%).

**Conclusions/Significance:**

JE imposes a heavy burden for families and society in Zhejiang Province. This study provides comprehensive empirical estimates of JE burden to increase awareness and strengthen knowledge of the public. These data may support provincial level public health decision making for prevention and control of JE. Ongoing surveillance for acute meningitis and encephalitis syndrome (AEMS) in sentinel hospitals, is needed to further refine estimates of JE burden.

## Author summary

Japanese encephalitis, an infection transmitted by mosquitos, is the most diagnosed viral encephalitis throughout Southeast Asia and the Western Pacific region. China has one of the world’s highest JE burdens. Although JE has been well-controlled in Zhejiang Province, it is associated with high mortality and disability which brings heavy burden and tremendous pain to families and society. However, specific data on costs and DALYs from the provincial level are absent. With this study, we merged multiple data sources and applied an expansion factor to estimate the total economic burden and disease burden of JE during 2013–2018 in Zhejiang Province. The total economic burden was estimated at US $12.01 million with an EF = 3. Of this, $8.32 million was due to direct economic cost and $3.69 million to indirect cost. The disease burden was 42.75 DALYs per million population according to the GBD 1990 methodology and 80.01 DALYs per million population according to the GBD 2010 methodology. The estimated data show a heavy burden of JE on the economy and health care system in Zhejiang Province. This quantification should improve awareness of health impact assessment and provide evidence for decision-making to further prevent and control JE.

## Introduction

Japanese encephalitis (JE), caused by Japanese encephalitis virus (JEV), is one of the most serious vector-borne viral encephalitis infections in Southeast Asia, the Western Pacific Region, and Northern Australia [1–2]. JEV is transmitted by mosquitos, mainly *Culex tritaeniorhynchus* in tropical and subtropical regions [3]. Approximately 3 billion people are exposed to the risk of JEV infection [4]. Newly updated estimates [5] indicate that about 100,308 JE cases and 25,125 deaths occurred in 2015 globally. Although symptomatic Japanese encephalitis is rare, and only approximately 1 in 250 infections results in severe clinical symptoms, the case fatality rate can be as high as 30% [2]. Permanent neurological or psychiatric sequelae can occur in 30%–50% of survivors, resulting in heavy health, social and economic burdens [2].

JE is a mandatory notifiable infectious disease in China. Proportion of JE cases reported by China to World Health Organization (WHO) varied between 15.27%-56.26% for 2013–2018 [6]. Zhejiang Province, which is located in southeast coastal China and has a subtropical monsoon climate, used to be a JE endemic area, with the highest incidence of 47.5/100,000 in 1967 [7]. With the widespread use of JE vaccines, the rapid development of economy and remarkable improvement of sanitary conditions, the JE incidence had decreased sharply after the 1970s. However, recent epidemiological surveillance show that the incidence is on the rise due to global warming and improvement of diagnosis level, as well as report awareness [8,9]. The proportion of adult cases is increasing [8,9]. JE usually peaks in July in Zhejiang Province. Most of the symptomatic cases have a rapid onset which begins with acute and severe clinical manifestation [1], such as clouding of consciousness, vomiting and often seizures, after which most patients are transferred to Grade A tertiary comprehensive public hospital to get high-level treatment. Thus, 95% or more JE cases are reported through web-based epidemiological surveillance platform by top public hospitals distributed in all the 11 cities in Zhejiang Province, which provides real-time data to support public health decisions.

However, passive surveillance usually underreports the total episodes which is far from enough to learn the complete information on JE burden. Misdiagnosis is common for JE for several reasons. Firstly, although WHO recommends testing for JEV-specific IgM antibody in a single sample of cerebrospinal fluid (CSF) or serum, using an IgM-capture ELISA [2], diagnostic tests are highly dependent on the sensitivity and specificity of detection kits, of which gold standard has not yet been established. Secondly, lower collection rate of clinical CSF samples, especially for mild cases. Thirdly, at least 20–30% of cases do not have detectable levels of IgM antibodies in CSF on admission and therefore are misclassified as negative for JE [10]. Although previous studies [11–15] have discussed the burden of JE, complete analysis on both economic and disease burden is limited. In addition, measurement of DALYs for a certain region based on local empirical data remain rare. Most studies prefer to conduct descriptive analysis with mortality or morbidity, and do not provide information on follow-up about the extent of disabilities due to JE. Considering the underreporting, the burden of JE is substantially underestimated. There is a great need to have a comprehensive situational analysis of JE burden over time to provide supportive data for immunization strategy, vector control, hospital-based active surveillance, and optimal allocation of health resources.

The objective of this study was to measure the economic and disease burden of JE in Zhejiang Province, 2013–2018. We estimated the economic costs of JE cases based on collected comprehensive primary data with sensitivity analysis, including direct cost, indirect cost, and the total cost. In addition, disease burden of JE was expressed in DALYs based on follow-up information of prognosis of illness. Parameters and assumptions were applied to construct extrapolation to the provincial level to provide evidence for health policy decisions.

## Method

### Ethics statement

The study protocol was approved by the ethics committee of Zhejiang Provincial Center for Disease Control and Prevention in 2018. Written informed consent was obtained from all participants or legal guardians before enrollment.

### Number of JE cases

JE is a notifiable communicable disease in China. Passive reporting is mandatory within 24 hours of diagnosis through National Notifiable Disease Registry System (NNDRS) according to the Law of the People’s Republic of China on the Prevention and Treatment of Infectious Diseases. Notification follows diagnosis by clinical and/or laboratory confirmation through detection of IgM in blood or cerebrospinal fluid samples. The data of morbidity and mortality of JE in Zhejiang Province were collected from NNDRS and case-based JE surveillance system (JESS) from 2013 to 2018, during which 149 cases were reported. Underreporting is a common problem in JE surveillance throughout the world [1,4,5]. To refine the estimates of the total number of JE cases, officially reported numbers can be adjusted for underreporting using an expansion factor (EF) [16–18], which can be calculated as the reciprocal of reporting ratio [16]. Campbell, et al [4] demonstrated the estimated global incidence of JE in 2011 was 1.8 per 100,000, of which only 10% were reported to WHO. Yin, et al [19,20] concluded from the Acute Meningitis and Encephalitis Syndrome Surveillance (AMES) during 2006–2009 in China that the number of unreported JE cases were about 2–3 times of the reported ones. Thus, in order to adjust for misdiagnosis and underreporting in the present study, we multiplied the actual reported JE cases by an EF of 3 according to the AMES study in China from 2006–2009.

### Subjects and field interviews

We considered all the 149 reported laboratory-confirmed JE cases through NNDRS and JESS during 2013–2018 as potential subjects in this study. Field investigation was conducted from September 2018 to April 2019. JE cases or their parents/spouse/guardians were interviewed through face-to-face or telephone by trained professional investigators using standard questionnaires to obtain basic characteristic information, treatment history, costs of various categories, workday loss, the prognosis of illness and evaluation of life quality.

### Economic burden of JE

#### Source of data-direct medical costs from hospitals’ billing system and interviews

Direct medical costs were defined as inpatient costs, outpatient costs and patient out-of-pocket medical costs. Inpatient and outpatient costs were reviewed through medical records in corresponding hospitals’ billing system in Zhejiang Province. The out-of-pocket medical cost was obtained from interviews using questionnaire.

#### Source of data-direct non-medical and indirect costs from interviews

Direct non-medical costs were defined as all expenses incurred due to the treatment, including nutritional supplement, transportation, accommodation, and employment costs for care givers, etc. Indirect costs were calculated using the human capital approach according to work-time loss caused by JE both for cases and their caregivers (the number of workday loss multiplied by the daily wages). Loss productivity was not calculated for children or students; rather for the corresponding care givers who asked for sick leave to take care of cases. Especially for those with severe sequelae who could not live independently, the indirect costs for caregivers contributed largely to the overall burden Because salary would change over time, to unify the evaluation criteria as well as for simplicity, all the severe disability or death-related income loss for cases and caregivers were calculated based on a 2-year period after the onset index date. In the event that participants chose not to disclose their income and in the absence of reliable data on average wages, we applied the standard average daily income from Zhejiang Provincial Bureau of Statistics as a proxy ($9960.40 /250 workdays = $39.84 per day) [21]. All the costs were expressed in 2018 US dollars (USD) using the 2018 average official exchange rate of 1 USD = 6.616 China Yuan, CNY according to the World Bank [22]. Considering for the currency inflation, a 3% discount rate was applied [16].

#### Extrapolation of the JE economic burden to the provincial level

Costs at the provincial level were estimated by multiplying the average cost per case by an estimate of the number of cases occurring in Zhejiang Province during 2013–2018, as well as taking under-reporting into consideration. The average cost per case and 95%CIs were computed with the percentile bootstrap method, with 10,000 replicates.

#### Associated risk factors of JE economic burden

Based on the prior knowledge, univariate risk factor analysis of JE economic burden was conducted using Wilcoxon rank-sum test or Kruskal-Wallis test. Data were entered and managed using EpiData (version 3.1), and data analyses were performed with SPSS (version 23). P-values less than 0.05 were considered significant.

#### Sensitivity analysis

To address the uncertainty in our estimates of the total economic burden of JE, deterministic sensitivity analyses were preformed to examine the effect of each parameter’s variation while keeping other parameters constant, including various risk factors for total economic cost. Each parameter was set to be the minimum and maximum observed to calculate the lowest and highest values. Calculations were performed using Microsoft Office Excel 2017.

### Disease burden of JE

The disease burden of JE in Zhejiang Province was measured in Disability-adjusted life years (DALYs) and expressed in provincial level based on incidence cases [23]. DALYs were the combination of years of life lost due to premature mortality (YLL) and years lived with disability (YLD) [24–25], and was calculated based on incidence, fatality rate, life expectancy at the age of death or onset, length of illness, and impact on quality of life (disability weight). We estimated the burden of JE using GBD (Global Burden of Disease) 1990 methodology [24–26] which took the age weight, treatment condition and time discounting rate (3%) into consideration for disability weight (Table 1) and expressed in the form of DALYs per million population. In addition, results using GBD 2010 methodology [27] which did not apply those social weightings were also presented due to the controversial application for those subjective choices. If the case had long-term sequalae both on cognitive impairment and neurological system, disability weight would be calculated as DW = 1-(1-DW_1_) *(1-DW_2_) assuming a multiplicative model [28]. Life expectancy at age of death or onset was obtained from the Coale–Demeny Model Life Table West (life expectancy at birth of 80 for males and 82.5 for females) to keep the consistency of the algorithm as well as for a better comparison with other studies [29]. The prognosis of illness was evaluated based on the questionnaire survey from patient interviews and mandatory follow-up assessment by general practitioners at 6 months after disease onset through JESS. Accounting for the length of illness for non-fatal cases, we calculated the time interval between the date of onset and the date of discharge from hospital for cases without sequela. However, for those with mild sequela which could be recovered in 6 months according to the follow-up health assessment, the duration of illness was considered to be 6 months [1]. For those with severe long-term sequalae who could not live independently, including cognitive impairment or/and neurological sequelae, we assumed the DW remained the same for the rest of their life expectancy from onset age.

**Table 1:** Age-specific disability weights for untreated and treated forms of sequelae included in the Global Burden of Disease Study for Japanese encephalitis [26,30].

		Untreated form					Treated form				
		*Age group (years)*					*Age group (years)*				
**Sequela**		**0–4**	**5–14**	**15–44**	**45–59**	**60+**	**0–4**	**5–14**	**15–44**	**45–59**	**60+**
**Japanese encephalitis**											
**1**	**Episodes**	0.616	0.616	0.613	0.613	0.613	0.616	0.616	0.613	0.613	0.613
**2**	**Cognitive impairment**	0.469	0.483	0.483	0.486	0.485	0.394	0.420	0.451	0.466	0.468
**3**	**Neurological sequelae**	0.388	0.388	0.388	0.397	0.468	0.334	0.334	0.334	0.337	0.390
**2+3**	**Both**	0.675	0.684	0.684	0.690	0.726	0.596	0.614	0.634	0.646	0.676

Note: age-specific disability weights in treated form were applied in this study as all the JE cases were reported from hospitals and had received treatment.

## Results

### Data collection and participants recruitment

Between September 2018 and April 2019, 61 laboratory-confirmed JE cases (or their parents/spouse/guardians) were available to be interviewed for the retrospective disease history, of whom 60 were valid for analysis and one case withdrew during the interview (S1 Table). These cases were reported from 7 cities in Zhejiang Province: 21 cases (35%) from Ningbo, 15 cases (25%) from Wenzhou, 7 cases (11.67%) each from Taizhou and Jinhua, 4 cases (6.67%) each from Jiaxing and Huzhou, and 2 cases (3.33%) from Lishui, which indeed reflect the different incidence levels of JE in different cities during 2013–2018 (*r* = 0.893, *P* value = 0.007). The basic information is described in Table 2. Among the 60 cases, 5 were fatal (8.33%) and 38 were male (63.33%). The onset age varied from 6 months old to 60 years old, with the average of 18.45 years old (95%CI: 14.45, 22.45). Half of the cases were migrants from other provinces in China. The occupation of participants was highly concentrated in students (from primary school to high school) and preschool children (30 cases, 50%), and blue-collar workers or farmers (17 cases, 28.33%). Sixteen cases (26.67%) had complete immunization records on JE through Immunization Information System in Zhejiang Province. Twenty-seven cases (45%) reported medical insurance, including primary public medical insurance and commercial insurance. Among the 55 non-fatal cases, 28 (50.91%) developed significant sequelae, including neurological sequelae and cognitive impairment. The length of inpatient duration for cases varied from 4 days to 759 days, with an average of 62.3 days and a median of 18.5 days. The workday loss for care givers varied from 5 to 860 person-days, with an average of 81.8 person-days and a median of 29 person-days.

**Table 2:** Average costs (mean with 95%CI) per JE case (in US$) according to different classification.

Type	No. of cases	Direct cost Medical	Direct cost Non-medical	Direct cost Total	Indirect cost	Total cost
**Prognosis of illness**						
Non-fatal cases without sequela	32	2531.24 (1980.32, 3132.03)^a^	321.09 (248, 388.98)^a^	2852.33 (2287.71, 3461.67)^a^	1025.91 (788.14, 1306.05)^a^	3878.24 (3197.58, 4607.76)^a^
Non-fatal cases with mild sequela	18	34868.50 (11564.10, 68231.65)^b^	1689.10 (572.42, 3304.40)^b^	36557.59 (12135.79, 70892.16)^b^	8362.26 (4462.58, 13700.60)^b^	44919.85 (16796.26, 84114.41)^b^
Non-fatal cases with severe sequela	5	42821.85 (23847.72, 59479.06)^b^	2786.77 (1436.07, 3991.49)^b^	45608.61 (25864.44, 62876.04)^b^	35857.44 (27889.12, 39841.60)^b^	81466.05 (64555.20, 98376.90)^b^
Fatal cases	5	26346.69 (6539.29, 49283.26)^b^	1472.84 (409.57, 2895.17)	27819.53 (6948.87, 51201.42)^b^	26638.04 (23315.28, 30191.88)^b^	54457.57 (30455.38, 81377.37)^b^
*P-value*		< 0.001	0.001	< 0.001	< 0.001	< 0.001
**Migration**						
Local	30	27861.77 (12236.78, 46965.10)	1630.62 (819.61, 2625.28)	29492.40 (13167.68, 49287.41)	10580.57 (5757.29, 15544.19)	40072.97 (19784.09, 63219.46)
Migrate	30	7287.40 (3928.74, 11817.09)	435.27 (319.59, 572.39)	7722.67 (4303.81, 12362.63)	5947.01 (2673.82, 9730.57)	13669.68 (7421.61, 21246.06)
*P-value*		0.079	0.101	0.101	0.141	0.076
**Residential location**						
Urban	17	32756.85 (7483.36, 67595.09)	1558.83 (385.43, 3216.62)	34315.68 (7832.51, 70519.47)	11800.10 (5398.66, 18572.56)	46115.78 (15396.74, 87325.62)
Rural	43	11572.30 (7024.09, 16788.02)	825.03 (517, 1182.28)	12397.33 (7596.82, 17835.28)	6865.71 (3711.77, 10514.81)	19263.05 (11467.06, 27871.23)
*P-value*		0.389	0.854	0.496	0.337	0.321
**Gender**						
Male	38	20561.31 (8125.31, 36697.75)	1111.14 (525.34, 1857.58)	21672.45 (8695.19, 38599.31)	8345.74 (4578.42, 12226.75)	30018.19 (13925.52, 48877.47)
Female	22	12415.70 (5759.85, 20176.86)	897.88 (396.70, 1472.36)	13313.58 (6225.15, 21718.31)	8122.24 (3272.51, 13609.33)	21435.82 (9795.44, 34374.90)
*P-value*		0.701	0.821	0.830	0.830	0.927
**Age group (years)**						
0–6	14	4995.50 (2781.93, 7696.60)^a^	335.66 (151.33, 553.94)^a^	5331.16 (3017.28, 8149.33)^a^	1932.31 (1201.08, 2811.23)^a^	7263.47 (4564.83, 10697.35)^a^
7–18	20	19145.38 (3522.97, 46073.51)^a^	715.46 (279.5, 1419.80)^a^	19860.84 (3837.57, 47444.29)	4077.77 (1042.20, 8075.28)^a^	23938.61 (4950.97, 55636.85)^a^
19-	26	23139.64 (12392.34, 36074.20)^a^	1652.62 (852.52, 2682.49)^b^	24792.26 (13471.18, 38660.12)^b^	14893.07 (9174.46, 20496.20)^b^	39685.33 (23503.39, 57006.44)^b^
*P-value*		0.049	0.003	0.026	0.002	0.005
**Occupation**						
Students	30	14147.72 (3584.20, 32164.54)	583.73 (257.31, 1060.88)^a^	14731.45 (3897.36, 33249.59)^a^	2984.12 (1095.70, 5766.14)^a^	17715.57 (5115.04, 39087.68)^a^
Worker/Farmer	17	12997.54 (6372.75, 20930.80)	847.51 (515.29, 1290.89)	13845.05 (6959.92, 21929.96)	12817.26 (6599.99, 19882.37)	26662.31 (15244.43, 40744.94)
Commercial	7	21021.04 (3692.29, 40334.27)	1545.42 (361.10, 2750.35)	22566.45 (4052.08, 43114.93)	13159.09 (4075.36, 23984.19)	35725.55 (8678.08, 66885.80)
Others^c^	6	43656.36 (12816.57, 88971.57)	3206.55 (536.09, 7157.91)^b^	46862.91 (13916.28, 96180.39)^b^	16049.46 (4362.63, 28194.35)^b^	62912.38 (18674.84, 123419.75)^b^
*P-value*		0.062	0.009	0.043	0.002	0.012
**Vaccination**						
Yes	16	8349.77 (2729.79, 16943.12)	602.66 (220.34, 1129.28)	8952.43 (2993.61, 18135.83)	4091.22 (1093.53, 8981.40)	13043.66 (4314.25, 26995.56)
No	34	25844.56 (12455.71, 43632.11)	1430.63 (722.95, 2269.59)	27275.19 (13170.62, 45814.15)	12132.91 (7662.27, 17155.56)	39408.09 (22163.85, 61158.53)
Unknown	10	4216.40 (1830.22, 8278.32)	369.27 (290.36, 450.44)	4585.67 (2191.41, 8654.57)	1784.89 (625.61, 3744.89)	6370.56 (3085.26, 12259.73)
P-value*		0.019	0.082	0.015	0.029	0.011
**Insurance**						
Yes	27	31881.54 (15116.59, 54507.68)	1749.43 (833.70, 2892.75)	33630.97 (16097.05, 56931.31)	11669.12 (6327.54, 17206.50)	45300.10 (23814.34, 72370.62)
No	15	7499.31 (2762.52, 15390.69)	402.55 (200.79, 681.38)	7901.86 (3034.22, 15970.68)	6932.43 (1877.99, 12764.85)	14834.29 (5122.90, 27007.70)
Unknown	18	4510.23 (2978.16, 6291.80)	483.54 (482.06, 486.50)	4993.77 (3462.5, 6775.33)	4265.25 (1268.74, 8094.41)	9259.02 (5152.58, 14051.57)
P-value*		0.032	0.164	0.032	0.131	0.057
**Overall**	60	17574.59 (9538.60, 27701.85)	1032.94 (615.43, 1586.96)	18607.53 (10199.97, 29293.33)	8263.79 (5398.60, 11636.93)	26871.32 (16107.34, 39162.00)

Note

^a,b^ Groups with different letters indicated significant difference through pairwise comparisons for polytomous variables, otherwise indicated no significant difference.

^c^ Including civil servant, technical staff, and the retiree. They were combined due to sparse data.

*Statistical tests were only conducted between those "Yes" and "No".

### Economic burden of JE in Zhejiang Province, 2013–2018

#### Estimated economic burden for the 60 JE cases

Table 2 shows a summary of five types of average economic costs for the 60 cases according to different classification. The overall average costs per case were respectively $18,607.53, $8,263.79 and $26,871.32 for direct economic cost, indirect economic cost, and the total economic cost, as shown in the last three columns in Table 2. Without considering the lifelong income loss caused by severe disability and death due to JE, direct medical cost was the largest proportion of the total costs ($17,574.59, 65.30%) for all the 60 cases. For non-fatal JE cases who completely recovered, the average costs per case were respectively $2,852.33, $1,025.91, and $3,878.24 for direct economic cost, indirect economic cost, and the total economic cost. For non-fatal cases with mild sequelae, the average costs per case were respectively $36,557.59, $8,362.26 and $44,919.85. For non-fatal cases with severe sequelae who could not live independently, the average costs per case were respectively $45,608.61, $35,857.44, and $81,466.05. For fatal cases, the three costs above were respectively $27,819.53, $26,638.04 and $54,457.57. Multiple pairwise comparisons indicated that significantly statistical differences were mainly derived from completely recovered cases and the other three types of cases on direct economic cost, indirect economic cost, and the total economic cost (*P* values all < 0.015), which suggested all types of economic burden were much lower if the JE case did not suffer sequalae. The proportions of direct economic cost in the total cost for fatal cases (51.08%) was significantly lower than those completely recovered (73.55%, *P* value = 0.026) or with mild sequelae (81.38%, *P* value = 0.016). Therefore, for cases with severe disabilities who could not live independently or died, 2-year period evaluated indirect economic cost accounted for a higher proportion (44.02%-48.92%), not to mention the lifelong influence for indirect economic burden.

#### Risk factors for economic burden

Univariate factor analysis indicted that prognosis of illness (which had been described above) and age group had significant influence on all the five types of costs. Compared with cases no more than 6 years old, cases over 18 years old suffered from heavier economic burden, especially for direct non-medical cost ($335.66 vs $1,652.62), indirect cost ($1,932.31 vs $14,893.07) and the total cost ($7,263.47 vs $39,685.33). Occupation, vaccination, and insurance had partial effects on the economic costs. Occupation significantly influenced almost all the five types of costs except for the direct medical costs. The difference mainly derived from students and the “other” population. Students or scattered children showed much lower costs. Regarding vaccination, cases without immunization history on JE had significant higher costs than those vaccinated, except for the direct non-medical costs. The average total cost for those unvaccinated was over 3 times the cost for those vaccinated ($39,408.09 vs $13,043.66). However, significant higher direct medical cost was observed in cases with insurance compare to those without ($31,881.53 vs $7,499.31), and insurance did not show any significant impact on the indirect cost. Other demographical factors, such as residential location, gender, and immigration, did not present any influence on various types of costs. Compared to the disposable income per capita in Zhejiang Province in 2018, 35.29% of urban cases and 53.49% of the rural cases had higher direct economic burden than the corresponding average annual disposable income ($8395.86 for urban residents and $4126.66 for rural residents) [21].

#### Total economic burden due to JE in Zhejiang Province

A total of 149 cases were reported to NNDRS and JESS from 2013 to 2018 in Zhejiang Province. Extrapolating the JE economic burden to the provincial level, if only the reported numbers were considered, the corresponding costs during 2013–2018 would be $2.77 million, $1.23 million, and $4 million for direct cost, indirect cost and total cost respectively. Given the situation of widespread underreporting of JE, an EF was applied. The total economic burden was estimated to be $12.01 million ($8.32 million for direct burden and $3.69 million for indirect burden) when EF = 3 according to the China report in 2011 and would be $40.04 million ($27.73 million for direct burden and $12.31 million for indirect burden) if EF = 10 according to the WHO report in 2011.

#### Sensitivity analysis

**Fig 1:**
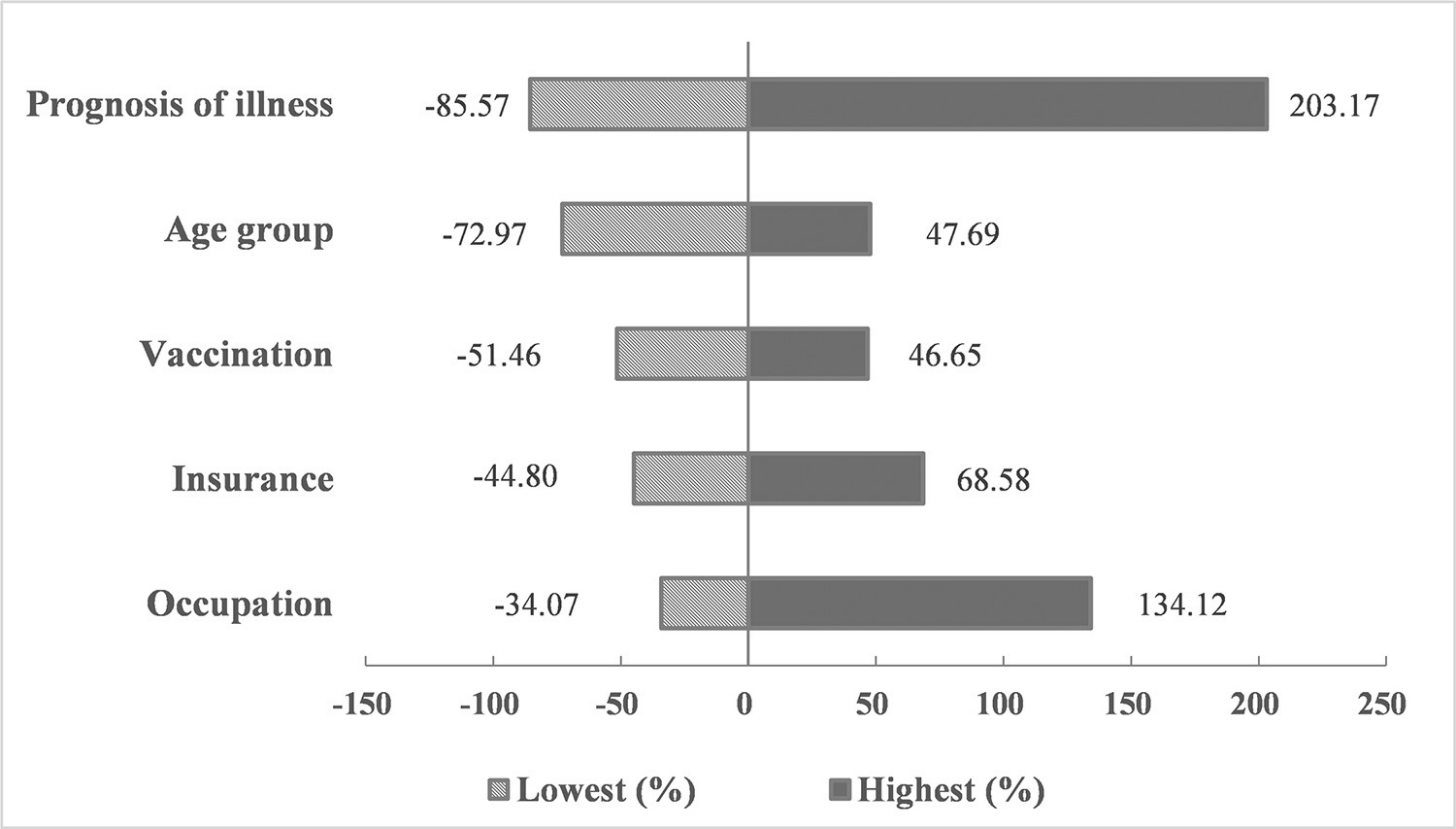
Variation in total economic burden of JE in Zhejiang Province according to extreme situation of significant risk factors included in the sensitivity analysis (2013–2018). Notes: The vertical line shows the point estimate for the total economic burden of JE ($12.01 million). The variation for each parameter corresponds to the value obtained from Table 2 for significant risk factors and were expressed in percentage change over the baseline.

All the significant risk factors of total economic burden were included in sensitivity analysis. Results were presented in the Tornado diagram (Fig 1) and corresponding estimated total economic burden under different situations in Zhejiang Province during 2013 to 2018 were presented in Table 3 (S1 Text). EF was set to be 3 considering under-reporting for the baseline calculation. Variation in any of these parameters resulted in overall economic burden varying from US$ 1.73–36.42 million. The greatest variation was due to the prognosis of illness (-85.57%-203.17%), followed by occupation (-34.07%-134.12%) and age (-72.97%-47.69%).

**Table 3:** The estimated total economic burden (in US$) of JE in Zhejiang Province, 2013–2018, under different extreme situation of corresponding significant risk factor.

	Lowest	Highest
**Prognosis of illness**	1,733,573.28	36,415,324.35
**Age group**	3,246,771.09	17,739,342.51
**Occupation**	7,918,859.79	28,121,833.86
**Vaccination**	5,830,516.02	17,615,416.23
**Insurance**	6,630,927.63	20,249,144.70

Note: EF was set to be 3 considering under-reporting in calculating total economic burden. Lowest/Highest denotes the lowest/highest total economic burden.

### Disease burden of JE in Zhejiang Province, 2013–2018

According to the National Surveillance Program of JE in China, mandatory follow-up health assessments conducted 6 months after onset by general practitioners in the community, combined with the information collected from field investigation for 60 cases, were evaluated to estimate the disease burden of JE in Zhejiang Province during 2013–2018. Among the reported 149 JE cases, 6 cases (4.03%) were lost to follow-up. Ninety-one cases (61.07%) completely recovered, 29 cases (19.46%) had mild sequelae which did not significantly influence their daily life, 11 cases (7.38%) remained severe sequelae and could not live independently, and 12 cases (8.05%) ended up with death.

Duration of illness was the key factor to calculate YLD and was significantly correlated with the length of time spent on caring cases by care givers (Spearman *r* = 0.932, *P*-value < 0.001) based on the collected data from 60 cases. Unlike total economic burden, only the prognosis of illness (Chi-square *χ*^*2*^ = 28.795, P-value < 0.001) and insurance condition (Mann-Whitney U = 117, *P-value* = 0.025) had significant impact on the duration of illness. Pairwise comparison indicated that completely recovered cases had a significantly shorter illness period (16.5 days) than the other three types of cases (all 100 + days, *P-value* < 0.01 for all). The average duration of illness for cases with insurance was almost 3 times that for those without (100.5 vs 35.4 days).

The total disease burden of JE in Zhejiang Province during 2013 to 2018 was 14.25 DALYs per million population, and the annual values for 2013–2018 were respectively 4.09, 3.50, 0.21, 1.15, 2.18, and 3.11 DALYs per million population, with the average to be 2.37. Year 2013 contributed the highest DALYs (28.70%), followed by Year 2014 (24.56%) and Year 2018 (16.63%). Among the total DALYs during 2013–2018, YLD represented about 66.56%, with the annual percentages respectively for 79.36%, 67.68%, 100%, 100%, 55.52% and 41.61% for 2013–2018. The GBD 2010 study dropped age weight and time discounting rate from the original 1994 definition of DALYs, which resulted in a higher weight of young children and old adults compared to young adults. Since JE cases in Zhejiang Province were mainly less than 15 years old, thus, the GBD 1990 methodology demonstrated a more conservative estimates of disease burden of JE compared to the GBD 2010 methodology (Table 4).

**Table 4:** Estimate disease burden of 149 reported Japanese encephalitis cases in Zhejiang Province per million population, 2013–2018.

	2013	2014	2015	2016	2017	2018	Average	Total
**GBD 1990 method**								
YLD	3.25	2.37	0.21	1.15	1.21	1.30	1.58	9.48
YLL	0.85	1.13	0.00	0.00	0.97	1.82	0.80	4.76
DALYs	4.09	3.50	0.21	1.15	2.18	3.11	2.37	14.25
**GBD 2010 method**								
YLD	6.63	4.25	0.35	2.08	2.14	2.44	2.97	17.89
YLL	1.50	2.40	0.00	0.00	1.69	3.19	1.47	8.78
DALYs	8.13	6.65	0.35	2.08	3.83	5.64	4.44	26.67

Note: YLD denotes Years Lost due to Disability. YLL denotes Years of Life Lost. DALYs denote Disability-Adjusted Life-Years. The GBD 1990 method refers to the original definition of DALYs proposed by Murray et al. in 1996 and used in most burden studies, which takes social weightings into consideration, including age weight, treatment condition and time discounting rate. The GBD 2010 method refers to an updated definition of DALYs used in the Global Burden of Disease 2010 study, in which age weight and time discounting rate were dropped from the estimates.

Similarly, EF = 3 was applied given under-reporting. The estimated total disease burden of JE in Zhejiang Province during 2013–2018 was 42.75 DALYs per million population (28.44 YLDs, 14.28 YLLs) using GBD 1990 methodology, assuming the age distribution, percentages of disability and death, severity of disability were the same as the reported 149 JE cases. However, if the GBD 2010 methodology was applied, the total disease burden would be 80.01 DALYs per million population (53.67 YLDs, 26.34 YLLs).

## Discussion

Japanese encephalitis remains a devastating disease with heavy economic and disease burden in Zhejiang Province. We used the overall number of JE cases between 2013 and 2018 to obtain stable estimate of the burden of JE, which we consider more useful reference value for policy purposes than an estimate for a specific year due to the annual variation of cases.

For economic burden of JE cases, we estimated that the overall average costs per case were respectively $18,607.53, $8,263.79, and $26,871.32 for direct economic cost, indirect economic cost, and the total economic cost. Two studies in China [11,13] had demonstrated comprehensive estimates of the economic burden of JE in their region within different time periods. Yin, et al [11] conducted an economic evaluation of JE vaccine in Guizhou Province, China and reviewed medical records of 68 JE cases, finding the average hospitalization medical cost was $682.6 in 2009. They further interviewed 16 cases, 12 of which had long-term sequelae, and found the cost of acute care was $1302 in total and the average cost for long-term sequelae care was $1782 per year. Another study [13] conducted in Gansu Province, China in 2006 interviewed 55 cases and demonstrated the median of direct cost were $359.64 (transformed in 2006 US$: 1:7.973 [22]) for fatal JE cases, and $864.04 for all cases. These 2 studies in China displayed lower estimates compared to our study, even considering for the annual inflation rate of 3%. Possible explanations are as follows: First, Zhejiang Province has significantly higher GDP per capita ($15,281) compared to Gansu Province ($4,645) and Guizhou Province ($6,446) in 2018 [21]; Second, the distribution of disease severity and onset age of cases (58.76% are > 15 years old in Gansu Province) involved in these studies are different, which have significant impact on the costs according to our analysis. Financial ability would influence the attitude to deal with sequelae, which further affects total costs. Some families would give up treatment due to poverty and lack of basic medical insurance; Third, the statistical description of costs is inconsistent, some use means and some use medians. Relevant studies abroad mainly focus on cost-effectiveness or cost-benefit analysis of JE vaccines, which may not give details of various types of economic burden for JE cases. For example, Vodicka [31] concluded from 40 suspected JE cases in Philippines during 2015–2017 that the JE-related hospitalization costs per case was $859, and the lost wages for caregivers was $398. Singh [32] investigated that the median of direct cost, indirect cost and total cost were respectively $216.48, $367.48, and $583.96 when transformed into 2014 US$ (1: 61.03) [22] from 120 JE cases in India. Touch [33] interviewed 61 cases in Cambodia during 2007–2009 and estimated the average direct cost of illness was $387.72-$519.94. Other estimates of economic cost per case in the region include Bali [34] (Indonesia, 2001–2002, direct medical cost: $311-$2124; direct non-medical cost + indirect cost: $156), Jiangsu Province [35] (China, 2007–2008, direct economic cost: $1859.30) and Shanghai Province [36] (China, 1998, cost for acute care: $1209, cost for long-term care per year: $121). Compared to these estimates listed above, our study demonstrated a higher economic burden per case, the possible explanations are as follows: First, costs from our study were calculated from primary data sources, including medical records for laboratory-confirmed JE cases and face-to-face patient or guardian interview, which better ensured the accuracy of data. Second, the coverage of costs from economic evaluation of JE vaccines might not be comprehensive. Some only focused on direct medical costs but expressed as direct economic burden. Some even just interviewed with clinicians to get the crude estimates. Some only concluded from the acute care costs and ignored the long-term sequelae costs which should be an important part for those with disabilities from our investigation. The paucity of data from health-seeking behavior prior to hospitalization, costs for long-term care and the workday loss for caregivers are possibly the most critical gaps in estimating the true economic burden of JE. Third, because most of the cost-effectiveness analysis concentrated on the benefits of JE vaccine which were mainly used for children, thus the economic costs were estimated from young age JE cases, which showed a significantly lower costs than adult cases in our study. Fourth, GDP plays an important role in economic burden, as well as the currency inflation.

In endemic areas, there are more studies on disease burden (i.e. morbidity and mortality) instead of studies on economic burden [37–42]. We estimated the DALYs of JE in Zhejiang Province during 2013–2018 was 80.01 per million population (53.67 YLDs, 26.34 YLLs) when EF = 3 was applied using GBD 2010 methodology. Previous studies always estimated DALYs of JE in the form of large endemic area. For example, Wadhwa, et al [42] estimated JE was associated with 491,797 DALYs in the Southeast Asia and 185,573 DALYs in the Western Pacific region in 2004. LaBeaud, et al [43] measured the global burden of arboviral diseases and concluded that 265,778–1,859,170 non-discounted DALYs (252,000–1,080,000 YLLs, 13,660–1,002,006 YLDs) were lost to JE in 2005 worldwide. If we converted these data into a comparable form and expressed them in units per million population according to the world population report (2005: 6,541,907 thousand) from the United Nations [44], the estimated DALYs in LaBeaud’s study was 40.63–284.19 DALYs per million and the proportion of YLLs can even be as high as 94.82% in 2005, which is significantly higher than our estimates during 2013–2018. The possible reason may be due to the high incidence and mortality rate in 2005, when JE vaccine had not been widely available in many endemic areas. Therefore, the prevention of JE and the prognosis of illness is challenging. In addition, EFs in our study was set to 3 according to Yin’s report in 2011[19], which maybe conservative. According to the reported JE cases data in Joint Reporting Form (JRF) from WHO [6], countries with the highest reported JE cases during 2013–2018 were respectively India (9732 cases with the proportion of 35.73%), China (7737 cases, 28.41%), and Nepal (2577 cases, 9.46%). Considering the fitted data from Campbell and Tran’s analytic modeling [4,5], the reported number of JE cases to WHO in 2015 might be 17 to 25-fold underestimated (4086 reported cases in reality vs 67,900 cases from Campbell’s estimate and 100,308 cases from Tran’s estimate). Therefore, the disease burden of JE was probably far more than what we collected and reported.

Risk factor analysis combined with sensitivity analysis indicated that the prognosis of illness has the biggest impact on the total economic burden, followed by occupation, age, insurance and vaccination. 50.91% developed significant sequelae among the 55 non-fatal cases in our study, which is comparable with other published articles [37,45,46]. The rehabilitation treatment for sequelae is long-term, periodical, as well as costly. Some studies [10,15,36] prefer to distinguish acute care with long-term care concerning in the direct medical cost estimate. Type of occupation determines the wages of workday loss, which affects the indirect economic cost estimate. Although JE vaccine has been introduced in Zhejiang Province since 1953 [9], the coverage rate did not reach 90% until 2008. Therefore, most adult cases during 2013–2018 did not get vaccinated. The influence of age on economic burden may be biased by vaccination. Previous studies [47–48] indicated that patients with insurance are likely to have clinical overtreatment and unnecessary use of expensive antiviral drugs, which is comparable to our study and leads to higher direct medical cost.

We do believe that we still underestimate the economic burden of JE. Several areas of uncertainty in our estimates deserve future attention. First, the true incidence and mortality rate is difficult to obtain. Annual active surveillance for acute meningitis and encephalitis syndrome (AEMS) in Grade A sentinel hospitals of all the 11 cities in Zhejiang Province is needed. Second, long-term sequelae of JE not only bring pain and sorrow to patient themselves, but also to their families, especially caregivers [49]. JE patients with severe sequelae, such as paralysis, dementia, or even both, need help with many aspects of life, including personal safety, mobility, daily activities, and social involvements. From our survey, mum/dad or spouse have to offer 24/7 service to look after severe cases who could not live independently due to JE, leading to decreased work productivity, and reduced wages. The long-term impact of household income loss is huge, as well as the psychological burden, which is hard to quantified as economic loss both for cases and caregivers. Third, long-term follow-up (> 5 years) is not available to evaluate the long-time prognostic outcome and extent of disability following JE. All the severe disability or death-related income loss for cases in our study were calculated based on a 2-year period after the onset index date, which made our estimates of indirect economic cost far more conservative. Fourth, due to data limitation, annual vector surveillance and control costs in Zhejiang Province were not included, as well as the corresponding time allocated by field personnel or sanitation workers. In addition, we acknowledge some limitations on risk factors analysis. Multiple regression model, such as ordinal logistic regression model, was not applied to control bias. However, small sample size with many multi-level polytomous categorical factors could lead to larger bias after fitting regression.

Future work will establish AEMS surveillance program, refine the corresponding estimates, keep up with more newly incident cases, have a better understanding of prognosis for extent disability and make advantage of some numerical scoring systems [50] to measure neurological disability and outcome due to JE.

## Conclusion

The total economic burden of JE during 2013–2018 in Zhejiang Province was estimated at US $12.01 million when accounting for under-reporting using an EF = 3. Of this, $8.32 million was due to direct cost and $3.69 million to indirect cost. The disease burden of JE was 42.75 DALYs per million population (28.44 YLD, 14.28 YLL) according to the GBD 1990 methodology and 80.01 DALYs per million population (53.67YLD, 26.34YLL) according to the GBD 2010 methodology. Our analysis provides estimates to public health policymakers in Zhejiang Province, helping to strengthen knowledge and raise awareness of the economic burden as well as disease burden of JE. These results can be used in health economic studies of novel vector control technology or JE immunization strategy programs. For the future, ongoing surveillance needs to be strengthened, including establishment of AEMS in sentinel hospitals, assessment of JE vaccination rate, evaluation of vaccine effectiveness and vector control. Continuous long-term follow-up information should be collected among JE survivors, and more clinical trials can be attempted on adult immunization due to aging trend of JE epidemiology.

## Supporting information

S1 STROBE Checklist (DOC)

S1 Table Details of economic burden for 60 JE cases in Zhejiang Province, 2013–2018. 60 laboratory confirmed JE cases (or their parents/spouse/guardians) were interviewed for the basic information, retrospective disease history, and the corresponding economic costs, combined with the billing information and medical records from hospitals.
(XLS)

S1 Text Example of calculation of sensitivity values. An example of how to calculate sensitivity values for risk factor—-Prognosis of illness.
(PDF)
