# Supplementary material for: Economic and disease burden of Japanese encephalitis in Zhejiang Province, 2013–2018
Source: PLoS Negl Trop Dis. 2021 Jun 21;15(6):e0009505. doi: 10.1371/journal.pntd.0009505 (PMC8248708; doi:10.1371/journal.pntd.0009505)
Supplement: S1 Text — An example of how to calculate sensitivity values for risk factor—-Prognosis of illness. (PDF) [file pntd.0009505.s003.pdf]

S1 Text. Supplementary Material. Example of calculation of sensitivity values. An example of how to calculate sensitivity values for risk factor——Prognosis of illness.

From Table 2, we can see "Prognosis of illness" has significant influence on total cost. Non-fatal cases without sequela have the lowest total cost of $3,878.24, and Non-fatal cases with severe sequela have the highest total cost of $81,466.05. Therefore, when we do sensitivity analysis for "Prognosis of illness", we assume that all the other factors stay the same and only the value for the studied factor——"Prognosis of illness" vary. So, the maximum value for total cost per case is $81,466.05 while the minimum value for the total cost is $3,878.24. According to Table 2, the overall average total cost is $ 26,871.32 (base value for total cost), then the highest variation in total cost for factor "Prognosis of illness" is

(81,466.05-26,871.32)/26,871.32*100%= 203.17%

while the lowest reduction is

(3,878.24-26,871.32)/26,871.32*100%= -85.57%.

For Table3, we set an EF=3, then the highest total economic burden of JE in Zhejiang Province during 2013-2018 = $81,466.05*149*3 =$36,415,324.35,

while the lowest value = $ 3,878.24*149*3=$1,733,573.28.
